# Highlights from Suicide Prevention Research in 2024

**DOI:** 10.1192/j.eurpsy.2025.725

**Published:** 2025-08-26

**Authors:** S. Tanyeri Kayahan, C. M. Platsa, M. Batković Šošo, M. Spasić Stojaković, S. Gomes Rodrigues, F. Kraxner

**Affiliations:** 1 Psychiatry, Republic of Türkiye Yalvaç State Hospital, Isparta, Türkiye; 2 Psychiatry, Barnet, Enfield and Haringey Mental Health NHS Trust, London, United Kingdom; 3 Psychiatry, General Hospital of Dubrovnik, Dubrovnik, Croatia; 4 Psychiatry, Theodor-Wenzel-Werk, Berlin, Germany; 5 Child and Adolescent Psychiatry, Centro Hospitalar Universitario de Santo Antonio, Porto, Portugal; 6 Psychiatry, Hospital Affoltern am Albis, Zürich, Switzerland

## Abstract

**Introduction:**

Suicide is a worldwide leading cause of mortality in youth, estimated to be the second leading cause of death for 10 to 24-year-olds. Increasing suicidality rates prompted worldwide national efforts to implement extensive suicide prevention plans. Those include crisis helplines, brief interventions such as safety planning and lethal means counseling, along with gatekeeper training and social prescribing.

**Objectives:**

This rapid review aimed to highlight the focus on suicide prevention-related research and review various preventive strategies reported throughout 2024.

**Methods:**

Participants from the European Psychiatric Association 2022 Summer School on “Focus on Suicide Behaviours - One Step Beyond” gathered and conducted a rapid, selective review of original research and review articles on the PubMed database using the search term “suicide prevention” with a time restriction to January-September 2024. Of 1529 results, 14 were selectively included depending on their specific discussion topics, such as suicide prevention in the perinatal period, social prescribing, social support system, and social network interventions, and crisis helplines. The main findings of the articles were summarized in specific brief sections.

**Results:**

Suicide being one of the leading causes of maternal mortality, the need for appropriate interventions for preventing maternal suicide was indicated (Gelabert *et al.* Matern Child Health 2024; 28(9) 1443-1453). Another topic of interest regarding the suicide rates among individuals without a mental disorder diagnosis being as high as one in five suicide attempters emphasized the scarcity of identifying this population’s risk factors (Oquendo *et al.* JAMA Psychiat 2024; 81(6) 572). A focus on crisis helplines as promising tools with a potential supportive impact on suicide prevention was obtained. Social prescribing, social support systems, and social network interventions were also identified as promising intervention strategies, particularly in adolescents (Dash *et al.* Front Public Healt*h* 2024; 12 1396614, Zheng *et al.* Prev Med Rep 2024; 39 102654). Gatekeeper training that aims to train community members to identify high-risk individuals and facilitate timely referrals and adequate support were found to have positive effects on gatekeeper behaviours (Spafford *et al.* Prev Sci 2024).

**Conclusions:**

Recent research in suicide prevention includes an emphasis on adolescent and perinatal suicidality. The evaluated interventions involve crisis helplines, social network interventions, and gatekeeper training. Meanwhile, this review was focused on specific aspects of suicide prevention; thus, its results are limited. Considering particular methodological and ethical challenges in studying the efficacy of suicide prevention interventions, further research encapsulating new approaches is needed.

**Disclosure of Interest:**

None Declared

